# Can Vitamin B12 Assist the Internalization of Antisense LNA Oligonucleotides into Bacteria?

**DOI:** 10.3390/antibiotics10040379

**Published:** 2021-04-03

**Authors:** Sara Pereira, Ruwei Yao, Mariana Gomes, Per Trolle Jørgensen, Jesper Wengel, Nuno Filipe Azevedo, Rita Sobral Santos

**Affiliations:** 1 Laboratory for Process Engineering, Environment, Biotechnology and Energy (LEPABE), Faculty of Engineering, University of Porto, R. Dr. Roberto Frias, 4200-465 Porto, Portugal; up201610825@fe.up.pt (S.P.); mggomes@fe.up.pt (M.G.); nazevedo@fe.up.pt (N.F.A.); 2 Biomolecular Nanoscale Engineering Center, Department of Physics, Chemistry and Pharmacy, University of Southern Denmark, Campusvej 55, 5230 Odense M, Denmark; ruweiy@kemi.dtu.dk (R.Y.); ptj@sdu.dk (P.T.J.); jwe@sdu.dk (J.W.)

**Keywords:** antibacterial drug, vitamin B_12_, antisense oligonucleotides, nucleic acid mimics, LNA, 2′OMe

## Abstract

The emergence of bacterial resistance to traditional small-molecule antibiotics is fueling the search for innovative strategies to treat infections. Inhibiting the expression of essential bacterial genes using antisense oligonucleotides (ASOs), particularly composed of nucleic acid mimics (NAMs), has emerged as a promising strategy. However, their efficiency depends on their association with vectors that can translocate the bacterial envelope. Vitamin B_12_ is among the largest molecules known to be taken up by bacteria and has very recently started to gain interest as a trojan-horse vector. Gapmers and steric blockers were evaluated as ASOs against *Escherichia coli* (*E. coli*). Both ASOs were successfully conjugated to B_12_ by copper-free azide-alkyne click-chemistry. The biological effect of the two conjugates was evaluated together with their intracellular localization in *E. coli*. Although not only B_12_ but also both B_12_-ASO conjugates interacted strongly with *E. coli*, they were mostly colocalized with the outer membrane. Only 6–9% were detected in the cytosol, which showed to be insufficient for bacterial growth inhibition. These results suggest that the internalization of B_12_-ASO conjugates is strongly affected by the low uptake rate of the B_12_ in *E. coli* and that further studies are needed before considering this strategy against biofilms in vivo.

## 1. Introduction

**Figure 1:**
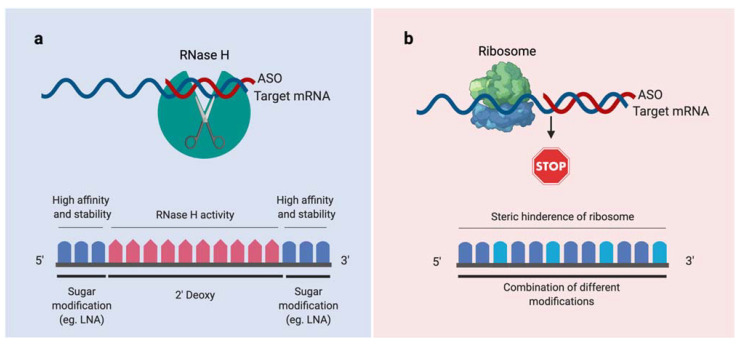
Different mechanisms that play a role in the modulation of the RNA function in bacteria. (**a**) Upon hybridization of a gapmer (red), the RNase H is recruited, and the target is degraded. (**b**) Steric hindrance of the ribosome caused by the hybridization between a steric blocker (red) and the complementary mRNA sequence. Figure created using BioRender.

The emergence of bacterial resistance to traditional antibiotics is considered a major threat in modern medicine [1,2]. Inevitably, innovative research focused on different antibacterial strategies is needed. Antisense oligonucleotides (ASOs) designed to inhibit bacterial gene expression have been gaining increased interest in recent years [3]. ASOs are especially interesting because even if bacteria develop a mutation that renders them resistant (one of the most common forms of resistance), the ASO can be easily redesigned to become an effective antibacterial drug again [4]. ASOs composed of nucleic acid mimics (NAMs), and in particular, locked nucleic acids (LNAs), possess improved target specificity, binding affinity, and resistance to exo- and endonucleases, compared to unmodified RNA or DNA [5,6], and have been successfully tested for clinical applications [7,8]. ASOs can be divided into two major categories, according to their mechanism of action: RNase H competent (or gapmers) and steric blockers (Figure 1). Gapmers are composed of DNA monomers that are typically flanked by LNA or other RNA-mimicking monomers. Upon hybridization of the gapmer to the target mRNA, the RNase H enzyme recognizes the DNA-mRNA heteroduplex and cleaves the target mRNA, leading to its degradation. Alternatively, the hybridization of steric blockers to the target mRNA simply physically blocks the access of the RNA polymerase to the target mRNA, thus directly inhibiting its translation [9,10,11]. There is only one study reporting the use of gapmers to target bacteria [11].

Nonetheless, the use of ASOs is limited by their inability to efficiently penetrate the complex envelope of bacteria. To overcome this burden, delivery vectors mostly focused on cell-penetrating peptides (CPPs) have been investigated. However, CPPs have been almost exclusively conjugated to neutral NAMs, such as peptide nucleic acids (PNAs) or phosphorodiamidate morpholino oligos (PMOs), which might present cytotoxicity and solubility issues [12,13,14]. Due to chemical conjugation difficulties, the vectorization of anionic ASOs with CPPs has been hampered. In a different approach, vitamin B_12_ (B_12_ or cobalamin), one of the largest molecules known to be taken up by bacteria, can be considered as a trojan-horse vector for neutral as well as anionic ASOs. The uptake system of B_12_ has been mostly studied in *E. coli*. [15]. *E. coli* uptakes B_12_ through the outer-membrane β-barrel protein BtuB in a TonB-dependent manner [16]. In the periplasm, BtuF binds to and delivers B_12_ to the ABC-type transporter BtuCD in the inner membrane, which, in turn, transports B_12_ into the cytoplasm [17,18].

Several functional groups are available for the modification of B_12_ to allow conjugation with ASOs, but not all modification sites are suitable to sustain their recognition and uptake [19]. Chromiński et al. described for the first time the synthesis of a clickable B_12_ derivate, possessing an azide functionality at the 5′ end [20]. This modification has already been tested for the copper-dependent conjugation of B_12_ with oligonucleotides, either composed of PNA or 2′OMe, mainly to inhibit genes that code for reporter proteins such as the red fluorescent protein (RFP) [6,20,21]. To our knowledge, there is only one study where a B_12_ conjugate was studied to decrease bacterial growth by inhibition of the essential gene *acpP* in *E. coli* using a PNA ASO [22]. This B_12_-PNA conjugate was only proved to inhibit *E. coli* growth in a very specific medium named Scarlett and Turner [22]. Under these conditions, even in the absence of B_12_ conjugates, bacteria only started growing after 48 h, while in other common minimal media, the exponential growth starts already after 5 h [23].

Occasionally, infections are associated with the formation of biofilms, adding an extra barrier for the use of antibacterial compounds [24]. ASOs were already shown to prevent biofilm formation and reduce mature biofilms, using either peptide nucleic acids (PNAs) or phosphorodiamidate morpholino oligomers (PMOs) as NAMs, conjugated to CPPs [25,26] but, to the best of our knowledge, never conjugated to B_12_. However, and because the cytosol is the ultimate target for these conjugates, it is important to first investigate their single-cell internalization.

In this study, we have investigated, for the first time, the internalization and inhibition efficiency of two different copper-free clicked conjugates, composed of vitamin B_12_ linked to LNA-based ASOs: a gapmer and a steric blocker. Both ASOs were designed to target the *acpP* gene in *E. coli*, which codes for an essential protein involved in fatty acid biosynthesis [27].

## 2. Results and Discussion

### 2.1. Conjugation of ASOs with Vitamin B_12_

**Figure 2:**
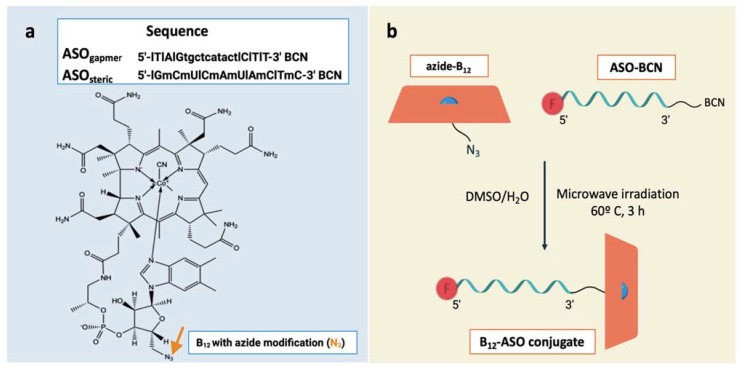
B_12_ and antisense oligonucleotides (ASOs) conjugation: sequences and structures. (**a**) Sequence of the ASO_gapmer_ and the ASO_steric_ (LNA nucleotide monomers are represented with upper case letters preceded by l, 2′OMe monomers are represented with upper case letters preceded by m, and DNA monomers are represented by lower case letters) and structure of 5′-end azide-modified B_12_ used in this study. The arrow points to the region of conjugation. (**b**) Schematic illustration of the synthesis of the B_12_-ASO conjugates through copper-free azide-alkyne chemistry.

In this study, two different kinds of LNA antisense oligonucleotides were designed and synthesized to target the *acpP* gene in *E. coli* (Figure 2a): an LNA/DNA gapmer (ASO_gapmer_) and an LNA/2′OMe steric blocker (ASO_steric_) [28,29]. While steric blockers have been widely tested in bacteria [4,27,30,31,32], gapmers have been mostly studied in mammalian cells [11]. As such, we intended to compare the potency of the different antisense mechanisms in *E. coli.* As the bacterial envelope poses a stringent barrier to the internalization of oligonucleotides, appropriate vectors must be applied for their transport into the bacterial cytosol. This is the first study documenting the use of B_12_ as a vector for LNA oligonucleotides. For the association of B_12_ to ASO_steric_ and ASO_gamper_, a copper-free ring-strain-promoted azide-alkyne coupling reaction was used (Figure 2b) [33]. This method proved to be efficient and resulted in satisfactory yields (Table S1). The increase in HPLC retention time for the B_12_-ASO_gamper_ and B_12_-ASO_steric_ compared with the ASO_gapmer_ and ASO_steric_ points toward efficient conjugation, confirmed by the obtained molecular masses, which are similar to the calculated theoretical values (Figure S1). The conjugation yields were determined as 70% and 97%, respectively, for B_12_-ASO_gamper_ and B_12_-ASO_steric_.

### 2.2. Bacterial Susceptibility Tests

**Figure 3:**
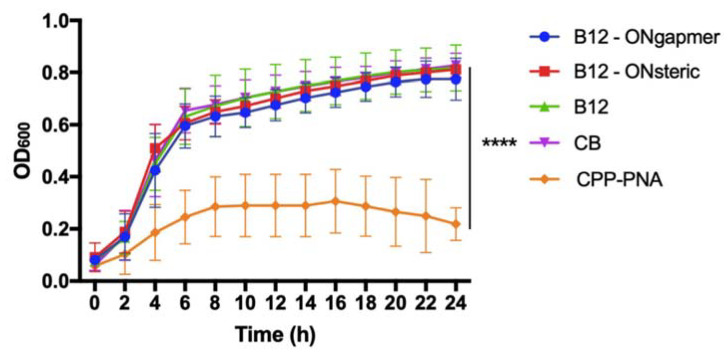
Growth of *E. coli* K12 in Davis minimal medium supplemented with B_12_-ASO_gapmer_, B_12_-ASO_steric,_ and B_12_ (at a concentration of 30 μM). CB represents the bacterial growth control in medium without any supplementation. Growth inhibition of *E. coli* K12 using a cell-penetrating peptide conjugated with an ASO composed of peptide nucleic acids (PNAs) (cell-penetrating peptides (CPP)-PNA) at a concentration of 30 μM is also shown. Results from three independent experiments (using duplicates in each) are presented as mean values and respective standard deviations. Statistical differences are indicated when appropriate in * (*p* ≤ 0.0001, ****).

Both ASOs were designed to recognize the *acpP* essential gene in *E. coli* and thus inhibit its expression. This should result in decreased *E. coli* viability, as long as the ASOs conjugated to B_12_ can efficiently penetrate the bacterial envelope. We investigated the ability of both conjugates (B_12_-ASO_gapmer_ and B_12_-ASO_steric_) to inhibit the growth of *E. coli* in Davis minimal medium at a concentration of 30 μM. This concentration was selected based on the good inhibition efficiency of a cell-penetrating peptide (CPP) conjugated with PNA, targeting the same gene (Figure 3, orange line). Our results show no inhibition using either conjugate composed of B_12_ at the same concentration (Figure 3).

In previous studies, PNA and 2′OMe steric blockers conjugated to B_12_ were able to decrease by 1-fold the expression of red fluorescence protein (RFP) in *E. coli* in Davis minimal medium [6,21]. However, to our knowledge, there is no other study including a regular growth control where B_12_-ASOs were investigated to kill bacteria, targeting an essential gene rather than a report protein. The only existing study uses a B_12_-PNA (ASO_steric_ targeting *acpP*) against *E. coli* in a particular medium where the control bacteria only starts growing after 48 h [22]. Nonetheless, the activity of this ASO sequence is already well established, as growth inhibition of *E. coli* K12 has been repeatedly reported using CPP-PNA [27,34,35], which was also confirmed herein. It is clear from the growth curves that the internalization occurs using the CPP as a vector for ASOs, as opposed to the B_12_ vector.

The lack of inhibitory effect of the *E. coli* K12 growth, observed with the conjugates synthesized in the present work, raises the question if the conjugates were efficiently internalized in the bacterial cells. In order to answer this question, location studies were performed next.

### 2.3. Evaluation of the Internalization of B_12_-ASOs

To examine the internalization of both conjugates in *E. coli* K12 and assess if association of the ASOs to the B_12_ could have hampered B_12_-promoted uptake, bacteria were observed under an epifluorescence microscope, after incubation with each of the Cy3-labeled conjugates or controls (B_12_, ASO_gapmer,_ and ASO_steric_).

**Figure 4:**
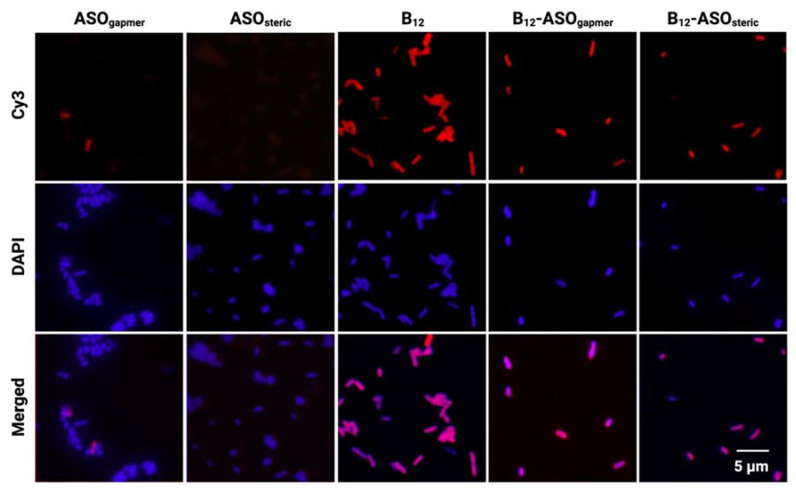
Interaction of Cy3-labeled ASOs, B_12,_ and B_12_ conjugates (concentration of 30 μM) with *E. coli* K12 after 4 h. Bacteria are counterstained with 4′,6′-diamidino-2-phenylindole (DAPI). Images are representative of three independent experiments (using duplicates in each). Scale bar represents 5 μm.

As expected, almost no fluorescent bacteria were detected when ASO_gapmer_ and ASO_steric_ were used alone (Figure 4–ASO_gapmer_ and ASO_steric_, Cy3 line). In contrast, it is clear that the conjugation of B_12_ to either ASO significantly increased the amount of fluorescently labeled *E. coli* K12, with all cells becoming fluorescent (Figure 4, B_12_-ASO_gapmer_ and B_12_-ASO_steric_). The same was observed for the B_12_ control (Figure 4, B_12_). Figure 4 shows images obtained at 30 μM, but a similar pattern was obtained for the lower concentration tested (15 μM, Figure S2).

These results point toward the B_12_-promoted association of the conjugates with the bacterial cells. However, the experimental distinction between membrane-associated and internalized molecules in bacteria remains a difficult task, given the small size of bacteria, which challenges the resolution limit of most standard equipment, including fluorescence microscopes [36].

**Figure 5:**
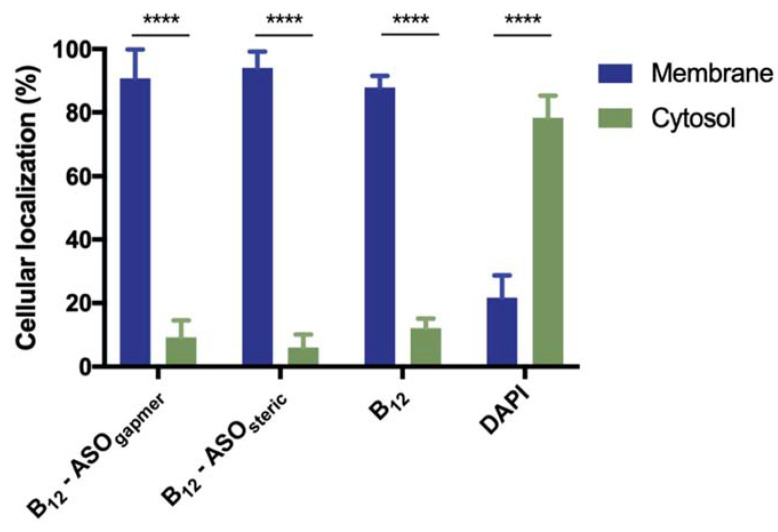
Cellular localization of B_12_ conjugates, B_12,_ and DAPI control in *E. coli* K12. B_12_ conjugates and B_12_ are mainly found on the OM, while the DAPI control is mostly associated with the cytosol. No significant differences were observed between the different internalized conjugates and between the conjugates and the B_12_ control (*p* > 0.05). Significant differences were observed between the membrane and cytosol-associated compounds (*p* ≤ 0.0001, ****). The fluorescence of each fraction present in the DAPI control is significantly different from the tested counterparts (*p* ≤ 0.0001). Results are presented as mean values and respective standard deviation from three independent assays (using duplicates in each).

Therefore, in an attempt to understand if the conjugates were internalized or membrane adhered, as well as to quantify their relative distribution, the bacterial cells were fractionated. A series of washing steps with a gradient of Triton X-100 concentrations was used to differentiate the membrane fraction from the cytosol [37]. These fractions were quantified using a fluorometer. Figure 5 clearly shows that only a small fraction of B_12_ and B_12_ conjugates completely penetrate the bacterial envelope into the cytosol (only 12%, 9%, and 6%, respectively, for the unconjugated B_12_, B_12_-ASO_gapmer,_ and B_12_-ASO_steric_), while more than 80% remain adhered to the membrane in all cases. The presence on the periplasm is not relevant (only ~3% of the conjugates were retained in this matrix, which was not statistically different from the cytosol. *p* > 0.05, Figure S3 [38]), which indicates that the BtuB at the outer membrane (OM) is likely the limiting factor for conjugate internalization into the cytosol. On the contrary, 4′,6′-diamidino-2-phenylindole (DAPI), a small and cell-permeant DNA intercalating dye, was majorly localized at the cytosol (Figure 5), as expected. Nonetheless, a small fraction was also present in the membrane (Figure 5), which can occur especially in non-fixed cells [39].

From the obtained fractionation results, it can be concluded that the microscopy fluorescence observed in Figure 4 is predominantly derived from conjugates associated with the OM of *E. coli* K12, rather than conjugates internalized in the cytosol, where the ASO would hybridize the *acpP* mRNA target. The inability of B_12_ to serve, in this study, as an efficient trojan-horse for the internalization of ASOs explains the lack of antimicrobial activity of the conjugates observed in Figure 3. It is possible that the uptake of B_12_ is strongly limited by the activity of BtuB, which is present in the OM.

The uptake of B_12_ is regulated by the expression/repression of the BtuB, the locus encoding for the B_12_ receptor [40]. B_12_ acts as a cofactor for methionine synthesis, necessary for growth [41]. In *E. coli*, it has been estimated that the methionine synthesis requires very low levels of B_12_ (20 molecules per cell) [42], while there are hundreds of thousands of mRNA copies of the *acpP* gene [43]. In our work, we have used a much higher concentration of B_12_ than the amount that *E. coli* needs for methionine synthesis. Hence, the difference between the amount of internalized B_12_ conjugates and the high amount of copies of the essential gene we aimed to inhibit may explain the lack of effectiveness of the conjugates.

In addition, it is also important to reflect on the future of this strategy, considering that in vivo, the number of internalized conjugates will probably be even lower since the host cells, as well as other bacteria from the microbiome, will compete for B_12_. Moreover, most in vivo infections are associated not with single-cell but with clustered cells organized in biofilms [44,45]. Therefore, the bioavailability of B_12_ conjugates may also be limited by interactions with the extracellular matrix. Nonetheless, genes encoding for virulent characteristics, such as the biofilm formation, are usually present in lower amounts of copies. Thus, it would be relevant to study the effect of B_12_-ASO conjugates targeting these genes in the future. In addition, the in vivo competition for B_12_ will favor bacteria with an improved affinity toward B_12_, as it has been found for some bacteria in the gut possessing an additional lipoprotein (BtuG) [46]. The use of B_12_ conjugates to target infections caused by such bacteria possessing BtuG could be considered in future biofilm studies.

## 3. Materials and Methods

### 3.1. Materials

All basic reagents used were purchased from commercial sources (Sigma-Aldrich, Søhus, Denmark) and used as received. Specific reagents and chemicals included LNA phosphoramidite monomers (Innovassynth Technologies, Maharashtra, India), DNA phosphoramidite monomers (Sigma-Aldrich, St Louis, MO, USA), 2′OMe phosphoramidite monomers, 3′-PT-amino-modifier C6, BCN *N*-hydroxysuccinimide ester (Glen Research, Sterling, VA, USA), DBCO-sulfo-Cy3 (Jena Bioscience, Jena, Germany) and Vitamin B_12_ (Carbosynth, Compton, UK).

### 3.2. Synthesis and Design of the ASOs

ASOs were designed to target the gene *acpP*, an essential gene coding for a protein involved in fatty acid biosynthesis. The particular *acpP* target region for the ASOs was selected based on previous studies [22,27,47]. Two different ASOs were synthesized: (i) an LNA/2′OMe chimera, designed for steric blocking (ASO_steric_), and (ii) an LNA/DNA chimera (ASO_gapmer_), designed to recruit RNase (sequences are represented in Figure 1a). Since LNA and 2′OMe substitutions increase the duplex stability, the ASO_steric_ was designed to be shorter than the ASO_gapmer_. ASOs were synthesized under anhydrous conditions using a PerSpective Biosystems Expedite 8909 nucleic acid synthesizer, as described elsewhere [48]. The synthesis was performed on a 1 µmol scale, using a 3′-PT-amino-modifier C6 support, with the following conditions: trichloroacetic acid in CH_2_Cl_2_ (3:97) as detritylation reagent, 0.25 M 4,5-dicyanoimidazole (DCI) in CH_3_CN as an activator, acetic anhydride in THF (9:91, *v*/*v*) as cap A solution, *N*-methylimidazole in THF (1:9, *v*/*v*) as cap B solution, and a thiolation solution containing 0.0225 M xanthan hydrate in pyridine/CH_3_CN (20:90, *v*/*v*). The coupling time was 4.6 min for both monomers. To obtain labeled ASOs, Cy3 phosphoramidite was added to the 5′ end in anhydrous CH_3_CN (0.1 M) and activated by tetrazole with a 15 min coupling time. The stepwise coupling yields were determined by the UV absorbance (at 500 nm) of dimethoxytrityl cations (DMT^+^) that were released after each coupling. The resulting ASOs were purified by reverse-phase HPLC (RP-HPLC), using a Waters System 600 HPLC equipment, equipped with a Waters XBridge BEH C18-column (5 µm, 100 nm × 19 mm). Their composition and purity (>85%) were confirmed by MALDI-TOF MS and ion-exchange HPLC analysis, respectively. Finally, the purified ASOs were labeled by reaction with BCN *N*-hydroxysuccinimide ester I in carbonate buffer (ASO:BCN = 1:2.5 equivalent) for 2 h. BCN labeled ASOs were desalted using NAP-10 Sephadex columns and purified by RP-HPLC. Their composition and purity (>95%) were confirmed by MALDI-TOF MS and analytical reverse-RP-HPLC, respectively. Concentrations of purified oligonucleotides were determined by UV absorption measurements at 600 nm.

### 3.3. Conjugation of ASOs with Vitamin B_12_

The 5′-azide-B_12_ was synthesized from commercially available vitamin B_12_ as described by Chromiński et al. [20]. Briefly, the 5′-hydroxy group of the B_12_ was transformed into a good leaving group (a mesyl group), and subsequently, azidation reaction provided the desired 5′-azide-B_12_. The 5′ position was chosen to avoid obstruction of both components of the conjugate [6]. The azide-B_12_ was isolated through precipitation. MS and NMR data were in accordance with the reported data [20].

Each Cy3-labeled ASO-BCN, dissolved in Milli-Q water, was added to a solution of azido-B_12_, dissolved in DMSO (ASO: azido-B_12_ = 1:2 equivalent). The resulting solution was transferred to a Biotage microwave reaction vial (0.5 mL) and sealed under a nitrogen atmosphere. The reaction was carried out on a Biotage Initiator microwave synthesizer at 60 °C for 3 h, whereupon all solvents were removed in vacuo, and the residue was re-dissolved in Milli-Q water (Figure 1b). Analytical RP-HPLC and MALDI-TOF MS were performed. The resulting solutions were de-salted by precipitation of the products by first adding an aqueous solution of sodium acetate (3 M, 15 μL) followed by the addition of cold ethanol (1 mL, 99% *w*/*w*; −20 °C). The resulting suspensions were stored at −20 °C for 1 h, and after centrifugation (16,000× *g*, 5 min, 4 °C), the supernatants were removed, and the pellet further washed with cold ethanol (2 × 1 mL; −20 °C), dried for 2 h and then dissolved in Milli-Q water (1 mL). Mass spectra of B_12_-ASO conjugates were recorded using MALDI-TOF MS, and the purity was confirmed by analytical RP-HPLC. Concentrations of purified conjugates were determined by ultraviolet absorbance at 260 nm.

The same procedure was conducted to obtain fluorescently labeled B_12_ (without ASO) where DBCO-sulfo-Cy3, instead of the Cy3-labeled ASOs, was conjugated to B_12_ through click-chemistry.

### 3.4. Bacterial Strain and Growth Conditions

*E. coli* K12 MG1655 was used in this study. To prepare the inoculum, the strain was grown overnight in tryptic soy broth (TSB) at 37 °C with shaking (180 rpm). To monitor both the inhibition of the *acpP* gene expression and the location of the conjugates, *E. coli* K12 was grown in Davis minimal medium at 37 °C with shaking (180 rpm) [49]. This medium lacks B_12_ in its composition, which was crucial to ensure that the internalized B_12_ comes from the control/conjugates incubated with bacteria [6,21].

### 3.5. Bacterial Susceptibility Tests

The inhibition of the expression of the essential *acpP* gene by the conjugates B_12_-ASO_gapmer_ and B_12_-ASO_steric_ was evaluated by monitoring the growth of *E. coli* K12, using a standard microdilution method. An overnight culture of *E. coli* K12 was diluted to an OD_600_ of 0.1 in fresh Davis minimum medium. These cell suspensions were added to wells of sterile 96-well plates and incubated with different concentrations of the tested compounds at 37 °C. The final concentration of the B_12_-ASOs and respective controls (B_12_, ASO_gapmer,_ and ASO_steric_) was 30 μM. As a control for the ASOs activity, the most well-studied vector-ASO conjugate was tested. In brief, a conjugate composed of the cell-penetrating peptide (KFF)_3_K and peptide nucleic acid (PNA) (Eurogentec, Seraing, Belgium), designed to hybridize with the same *acpP* sequence was tested in the same conditions as the B_12_ conjugates. The absorbance at 600 nm was determined on a BMGLabtech SPECTROstar Nano microplate reader for 24 h. *E. coli* in medium without any added compound was used as control (CB). Experiments were performed in three independent biological replicates.

### 3.6. Evaluation of the Internalization of B_12_-ASOs by Epifluorescence Microscopy

To evaluate the extent of internalized B_12_-ASO conjugates, compared with the ASOs alone, we used epifluorescence microscopy. An overnight culture of *E. coli* K12 was diluted to an OD_600_ of 0.1 in fresh Davis minimum medium. The B_12_-ASO_gapmer_, B_12_-ASO_steric_, B_12_, ASO_gapmer,_ and ASO_steric_ (all Cy3-labeled) were diluted in sterile distilled H_2_O and added to the bacterial suspension to a final concentration of 15 and 30 μM per test tube. After 4 h incubation, tubes were centrifuged (3000× *g*, 10 min), and the pellets were resuspended in sterile distilled H_2_O. To label the bacterial cytosol, 4′,6′-diamidino-2-phenylindole (DAPI) staining was used. Staining was performed by placing a drop of DAPI (0.5 μg/mL) on top of the dried sample for 5 min. The samples were visualized on a Nikon Eclipse T*i* SR epifluorescence microscope using a Nikon Plan-Apo 100X objective. Ten pictures of each sample were taken randomly, covering all the areas of the sample, using a QImaging Retiga R1 monochromatic camera, and processed with the NIS-Elements Advanced Research. The exposure time and the excitation intensity were maintained throughout the experiments. A G-2A longpass filter (excitation: 535 nm; emission: 580 nm) and a DAPI bandpass filter (excitation: 375 nm; emission: 460 nm) were used. The images obtained using both filters were merged using the Fiji software. Three repeated samples were analyzed for each condition, and three independent experiments were performed.

### 3.7. Evaluation of the Internalization of B_12_-ASOs by Bacterial Fractionation

To determine the location of the conjugates in the *E. coli* K12 cells visualized in the previous section and investigate if the observed fluorescence could derive from association to the bacterial envelope, as opposed to intracellular hybridization, the cells were fractionated, and the fluorescence of the outer-membrane fraction and periplasm vs. the fluorescence of the cytosol fraction was measured.

**Figure 6:**
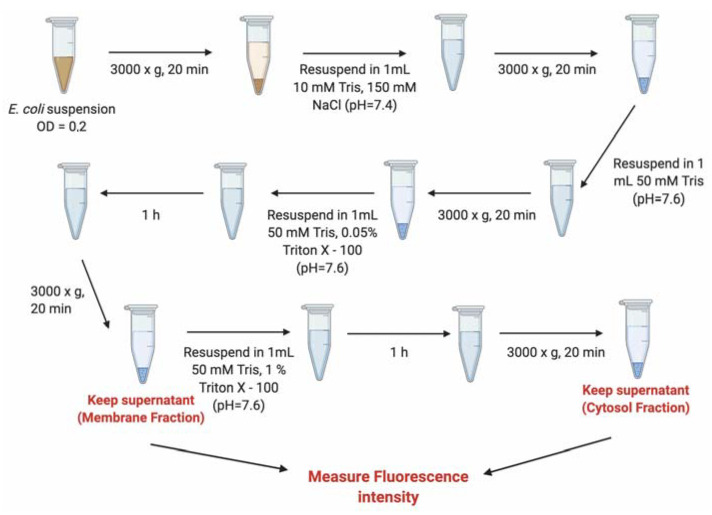
Fractionation protocol adapted from Bandula et al. [50]. A series of washing steps with a Triton X-100 gradient allows the isolation of the membrane and the cytosol fractions.

An overnight inoculum of *E. coli* K12 was diluted to an OD_600_ of 0.1 and grown in Davis minimal medium in the presence of 30 μM of B_12_-ASO_gapmer_, B_12_-ASO_steric,_ and B_12_, (all Cy3-labeled) for 4 h. Thereafter, a fractionation protocol (Figure 6) adapted from Banbula et al. [50] was followed. In brief, bacteria were centrifuged (3000× *g*, 20 min), resuspended in 10 mM Tris-150 mM NaCl (pH 7.4), and washed with 50 mM Tris (pH 7.6). To obtain the fraction associated with the outer-membrane (membrane fraction), bacteria were centrifuged (3000× *g*, 20 min) and resuspended in 50 mM Tris buffer solution containing 0.05% Triton X-100 (pH 7.6), for 1 h at room temperature (RT). After new centrifugation (same conditions), the Cy3 fluorescence intensity of the supernatant (membrane fraction) was measured with a fluorometer (BMGLabtech Fluorostar Omega), using 550 nm excitation and 570 nm emission filters. To obtain the fraction associated with the cytosol, the pellet was resuspended in a more astringent buffer containing 50 mM Tris 1% Triton X-100 (pH 7.6), for 1 h at RT. The supernatant resultant from the last centrifugation was removed, and the Cy3 fluorescence of the cytosol (cytosol fraction) was measured. As a control, the same fractionation protocol was applied to bacteria stained only with DAPI, and the fluorescence of the membrane and cytosol fractions was measured using 375 nm excitation and 460 nm emission filters.

### 3.8. Statistical Analysis

For the evaluation of the statistical significance, the two-way analysis of variance test (ANOVA) followed by Sydak’s multiple comparisons was used. A *p*-value of *p* ≤ 0.05 was considered statistically significant.

## 4. Conclusions

In summary, this innovative investigation discloses the challenges that need to be overcome before B_12_ -mediated ASO internalization is considered realistic toward tackling the challenge of antimicrobial resistance. This strategy is based on the uptake of the micronutrient B_12,_ which seems to be insufficient to act as an efficient trojan-horse for ASOs designed to inhibit the expression of an essential bacterial gene. In the future, it would be relevant to assess the concentration of internalized ASOs needed to efficiently knock down bacterial genes and inhibit bacterial growth. In addition, improving the bioavailability of vitamin B_12_ by modifying the conjugates and choosing better adapted bacterial targets would be important for successful translation from in vitro to in vivo application.

## Data Availability

The data presented in this study are available in the article and in the supplementary material.

## Supplementary Materials

The following are available online at https://www.mdpi.com/article/10.3390/antibiotics10040379/s1, Figure S1: Analytic RP-HPLC trace and MALDI-MS on B_12_-ASOgapmer and B_12_-ASO_steric_. The top panel shows the retention time (in minutes) of the B_12_ conjugates, and the bottom panel shows the mass (m/z) of each B_12_ conjugate.; Figure S2: Interaction of Cy3 labeled ASOs, B_12,_ and B_12_ conjugates with *E. coli* K12, after 4 h incubation at a concentration of 15 μM. Bacteria are counterstained with DAPI. Images are representative of three independent experiments (using duplicates in each). Scale bar represents 5 μm; Figure S3: Most of the conjugated (B_12_-ASO_steric_) and unconjugated B_12_ (B_12_) are prevented from internalization into *E. coli* cytosol since they remain at the outer-membrane; the percentages in the periplasm are only residual. The isolation of the periplasm was performed after the isolation of the OM fraction and was based on the fractionation protocol by Malherbe et al. 2019. *E. coli* cells were washed in spheroplast buffer (0.1 M Tris-NaCl, 500 mM sucrose, 0.5 mM EDTA, pH 8.0) followed by resuspension in distilled water and incubation for 15 s on ice. The osmotic shock occurred after the addition of MgSO_4_ (final concentration 20 mM). DAPI was used as a control, majorly localizing at the cytosol, as expected. Statistical differences are indicated when appropriate in * (*p* ≤ 0.0001, ****); Table S1: Characterization of the synthesized conjugates, including their HPLC retention times (t_R_) and molecular masses, as well as the yield of the respective conjugation reactions.
