# Supplementary material for: Can Vitamin B12 Assist the Internalization of Antisense LNA Oligonucleotides into Bacteria?
Source: Antibiotics (Basel). 2021 Apr 3;10(4):379. doi: 10.3390/antibiotics10040379 (PMC8065541; doi:10.3390/antibiotics10040379)
Supplement: Supplementary file 1 [file antibiotics-10-00379-s001.pdf]

Supplementary Materials

Table S1. Characterization of the synthesized conjugates, including their HPLC retention times (t_R) and molecular masses, as well as the yield of the respective conjugation reactions.

**Conjugate**	**HPLC t_R [min] (Analytical RP-HPLC)**	**Molecular mass [g/mol] (MALDI-TOF MS)**		**Yield [%]**
		**Theoretical**	**Experimental**	
B_{12}-ASO$_{gapmer}$	17.8	7504.1	7505.1	70.1
B_{12}-ASO$_{steric}$	18.1	5660.1	5658.7	97.4

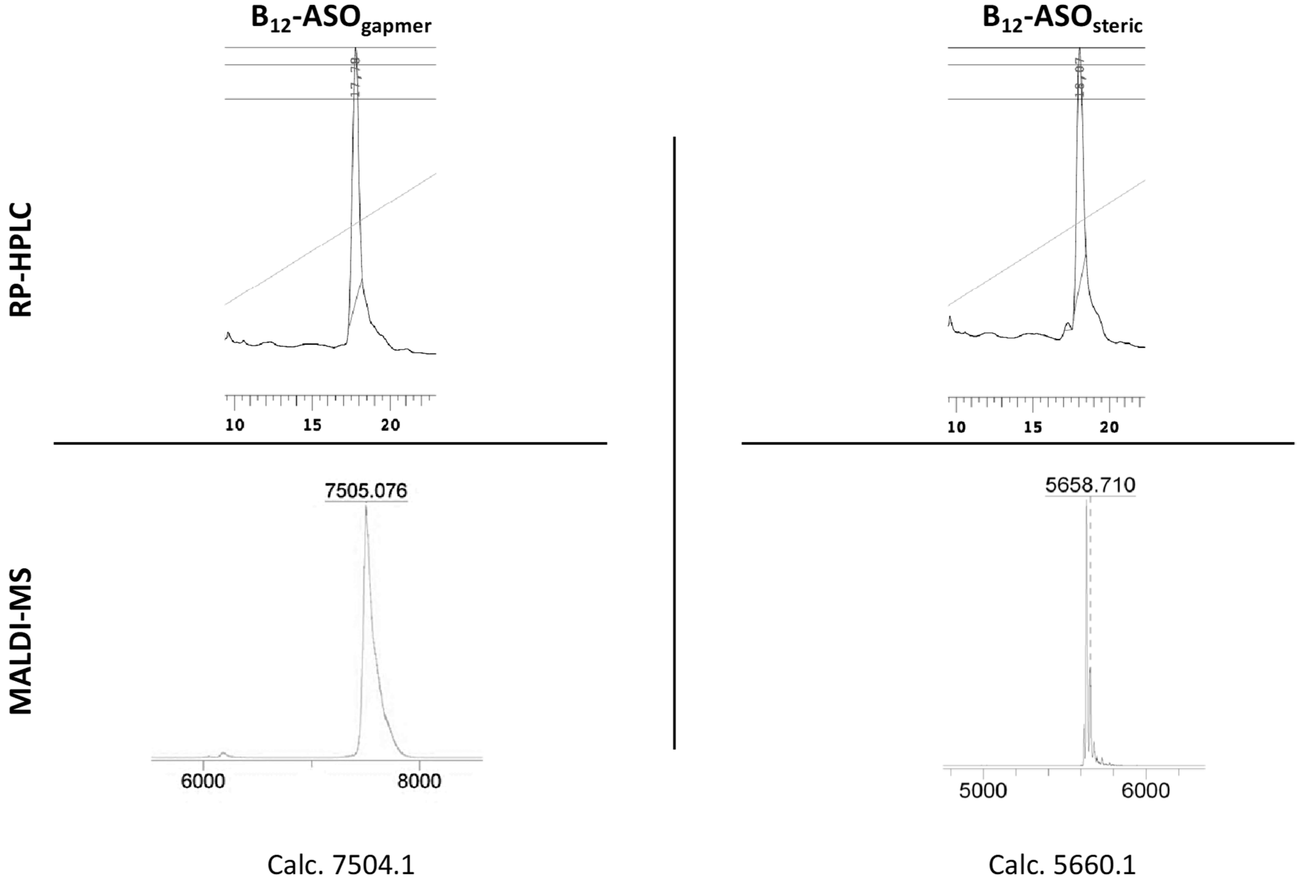

Figure S1. Analytic RP-HPLC trace and MALDI-MS on B_{12}-ASOgapmer and B12-ASO$_{steric}$. The top panel shows the retention time (in minutes) of the B_{12} conjugates and the bottom panel shows the mass (m/z) of each B_{12} conjugate.

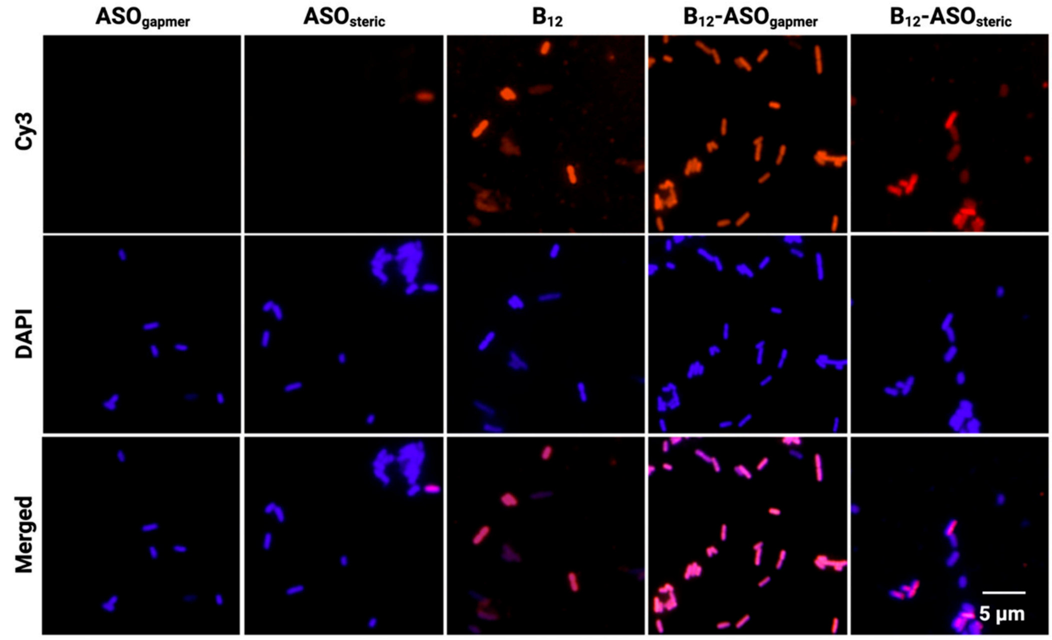

Figure S2. Interaction of Cy3 labeled ASOs, B_{12} and B_{12} conjugates with *E. coli* K12, after 4 h incubation at a concentration of 15 µM. Bacteria are counterstained with DAPI. Images are representative of three independent experiments (using duplicates in each). Scale bar represents 5 µm.

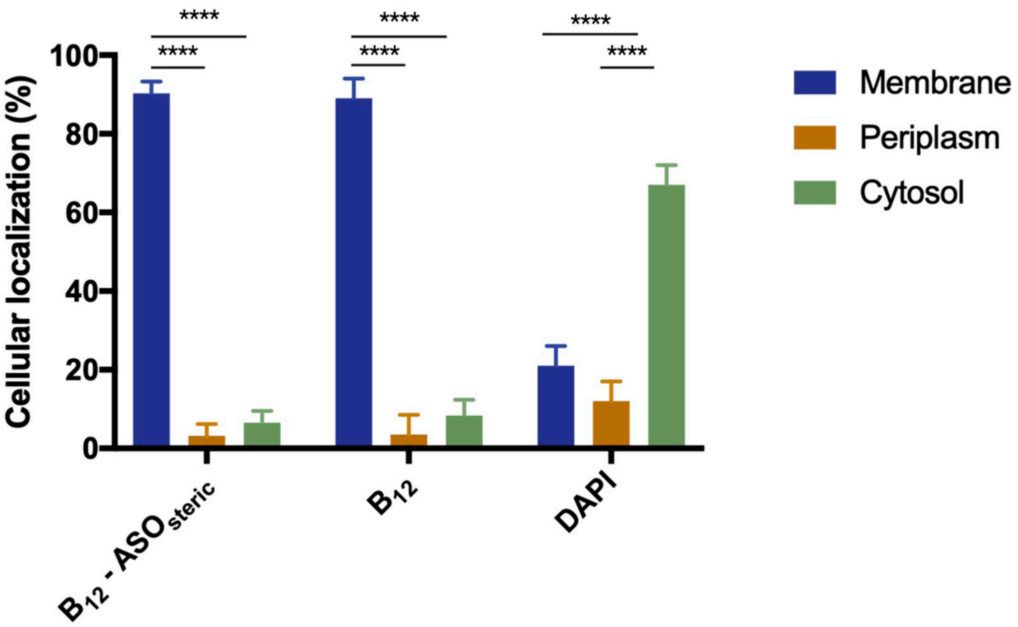

Figure S3. Most of the conjugated (B_{12}-ASO_{steric}) and unconjugated B_{12} (B_{12}) are prevented from internalization into *E. coli* cytosol since they remain at the outer-membrane; the percentages in the periplasm are only residual. The isolation of the periplasm was performed after the isolation of the OM fraction and was based on the fractionation protocol by Malherbe et al. 2019. *E. coli* cells were washed in spheroplast buffer (0.1 M Tris-NaCl, 500 mM sucrose, 0.5 mM EDTA, pH 8.0) followed by a resuspension in distilled water and incubation for 15 s on ice. The osmotic shock occurred after the addition of $MgSO_4$ (final concentration 20 mM). DAPI was used as a control, majorly localizing at the cytosol, as expected. Statistical differences are indicated when appropriate in * ($p \leq 0.0001$, ****). The fluorescence of each fraction present in the DAPI control is significantly different from the tested counterparts ($p \leq 0.0001$). Results are presented as mean values and respective standard deviation from three independent assay (using duplicates in each).
